# Risk factors for long-term survival in patients with ypN+ M0 rectal cancer after radical anterior resection

**DOI:** 10.1186/s12876-022-02226-9

**Published:** 2022-03-26

**Authors:** Marcin Zeman, Władysław Skałba, Piotr Szymański, Grzegorz Hadasik, Dmytro Żaworonkow, Dominik A. Walczak, Agnieszka Czarniecka

**Affiliations:** The Oncologic and Reconstructive Surgery Clinic, Maria Sklodowska-Curie National Research Institute of Oncology, Gliwice Branch, Wybrzeze Armii Krajowej 15, 44-100 Gliwice, Poland

**Keywords:** Angiotensin-converting enzyme inhibitors, Lymph node metastasis, Lymph node yield, Negative lymph node count, Rectal cancer, Renin-angiotensin system

## Abstract

**Background:**

Regional lymph node metastases are the main adverse prognostic factor in patients with rectal cancer without distant metastases. There are discrepancies, however, regarding additional risk factors in the group of ypN + M0 patients. The purpose of the study was to assess clinical and pathological factors affecting long-term oncological outcomes in the group of ypN + M0 patients after radical rectal anterior resection.

**Methods:**

112 patients with ypN + M0 rectal cancer after neoadjuvant therapy and radical anterior resection were subject to a retrospective analysis. The effect of potential factors on survival was assessed with the use of Kaplan–Meier curves together with a log-rank test and multiple factor Cox proportional hazards model.

**Results:**

In the multiple factor Cox analysis, adverse factors affecting disease-free survival (DFS) were: the use of angiotensin-converting enzyme inhibitors (ACEIs) (hazard ratio HR: 3.11, 95% CI 1.01–9.56, *p* = 0.047), presence of perineural invasion (HR: 7.27, 95% CI 2.74–19.3, *p* < 0.001) and occurrence of postoperative complications (HR: 6.79, 95% CI 2.09–22.11, *p* = 0.001), while a positive factor was the negative lymph node (NLN) count > 7 (HR: 0.33, 95% CI 0.12–0.88, *p* = 0.026). In the disease-specific survival (DSS) analysis, an adverse factor was the use of ACEIs (HR: 4.275, 95% CI 1.44–12.694, *p* = 0.009), while a positive effect was caused by NLN > 5 (HR: 0.22, 95% CI 0.082–0.586, *p* = 0.002).

**Conclusions:**

The use of ACEIs may have a negative effect on long-term treatment outcomes in patients with ypN + M0 rectal cancer. In this group of patients, the NLN count seems to be an important prognostic factor, as well.

**Supplementary Information:**

The online version contains supplementary material available at 10.1186/s12876-022-02226-9.

## Background

Regional lymph node metastases are the main adverse prognostic factor in patients with colorectal cancer without distant metastases [1]. However, reports on the risk factor of long-term survival in the group ypN + are not consistent. So far, the effect of comorbidities and the medication used therein, such as metformin or renin-angiotensin system inhibitors (RASIs), on the treatment outcomes has not been clarified [2, 3]. There are few publications on this issue. It has also been shown that postoperative complications, especially anastomotic leakage (AL) after rectal anterior resection (AR) may have a significant effect on survival, The results of analyses, however, are not conclusive [4, 5]. Knowing the above-mentioned, at least partially modifiable, factors could create a relatively easy possibility of affecting long-term treatment outcomes of patients with rectal cancer.

It turns out that the assessment of nodal staging according to the TNM classification does not clearly stratify subjects after neoadjuvant therapy with regard to long-term survival [6]. Therefore, attempts have been made to assess other factors, such as lymph node ratio (LNR), log odds of positive nodes (LODDS), positive lymph nodes (PLN), or negative lymph nodes (NLN). Their prognostic value, however, has not been finally established [1, 7]. A separate issue is the minimum lymph node yield (LNY), owing to which underestimation of nodal staging may be avoided. Some authors deny the adverse effect of low LNY on survival, and even suggest that it is related to a good response to neoadjuvant therapy [8].

The purpose of the study was a retrospective assessment of clinical and pathological factors affecting long-term oncological outcomes in patients with rectal cancer at ypN + M0, after neoadjuvant therapy and radical (R0) AR.

## Methods

### Patients

**Fig. 1:**
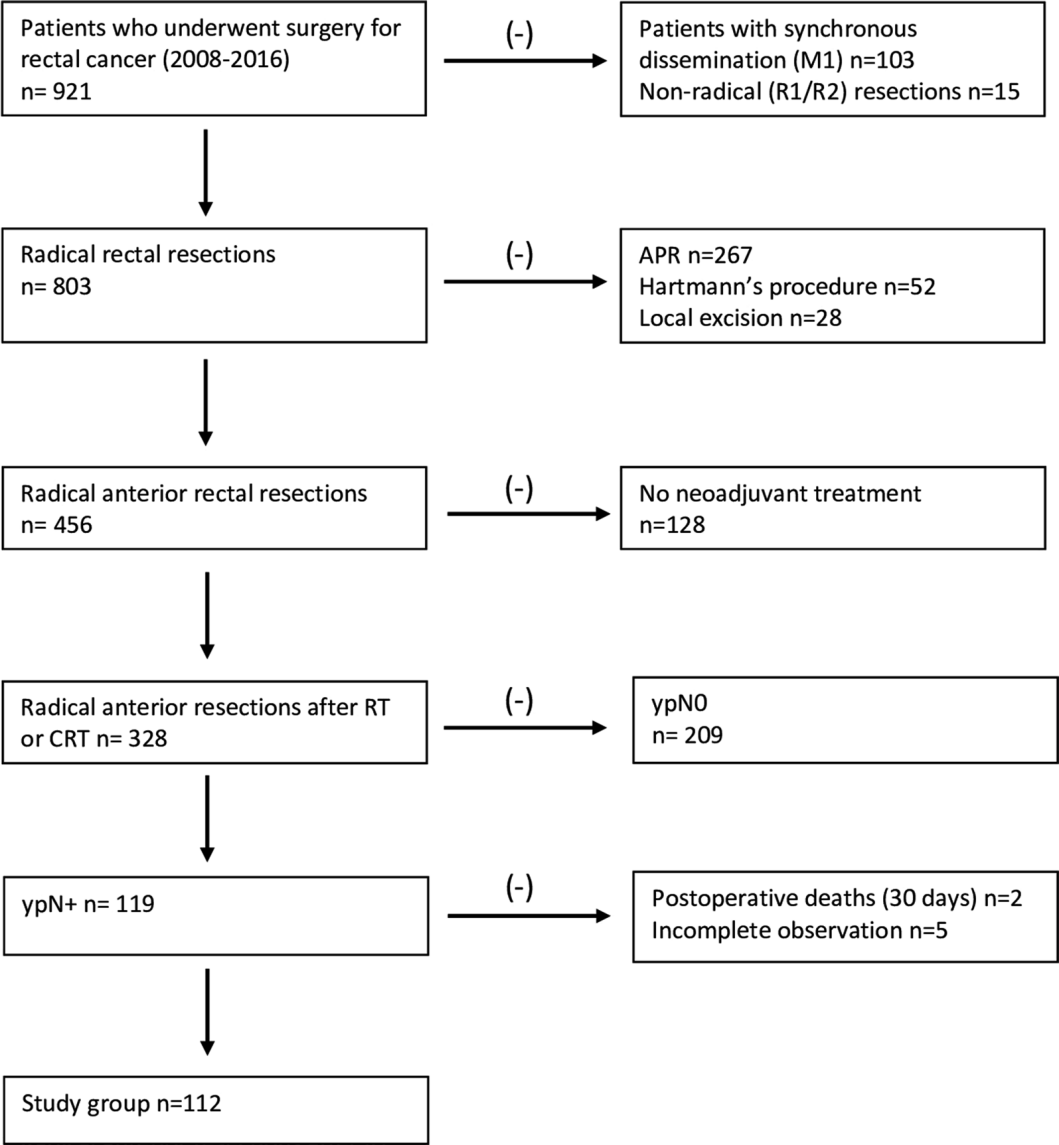
Process of study group formation

A retrospective analysis was performed on 112 patients with ypN + M0 rectal cancer post neoadjuvant therapy and radical (R0) AR, treated at the National Research Institute of Oncology in Gliwice in 2008–2016. Patients meeting the following criteria were included in the analysis: histopathological diagnosis of adenocarcinoma (up to 15 cm from the rectal margin), past neoadjuvant treatment, past radical (R0) rectal anterior resection, presence of regional lymph node metastases in a postoperative histopathological examination (ypN+), no synchronous distant metastases. Exclusion criteria: no neoadjuvant treatment, abdominoperineal rectal resection, Hartmann’s procedure, local resection of tumour, non-radical resection (R1 or R2), no regional lymph node metastases in a postoperative histopathological examination (ypN0), presence of synchronous distant metastases, death in the postoperative period (within 30 days). In addition, subjects with yN1c staging were excluded from the part of analysis regarding the assessment of nodal staging parameters, where it was necessary to provide the metastatic nodal count (LNR, LODDS, PLN), since it was not possible to perform a retrospective assessment of the tumour deposits count in all findings of the histopathological examinations. The process of the study group formation is presented on the chart in Fig. 1.

Patient characteristics is presented in Table 1. Comorbidities were assessed separately, as well as on the basis of the Charlson comorbidity index (CCI) [9]. Distant metastases were considered synchronous if occurred up to 3 months after surgery.

**Table 1:** Patient characteristics

Variable	n (%) mean (SD)*
Sex	
Females	42 (37.5)
Males	70 (62.5)
Age (years)	62.21 (10.32)*
BMI (kg/m^2^)	26.64 (5.05)*
CAD	5 (4.5)
AH	45 (40.2)
Diabetes mellitus	18 (16.1)
CCI > 2	25 (22.3)
Alpha blockers	3 (2.7)
Beta blockers	26 (23.2)
ACEIs	19 (17)
Calcium channel blockers	14 (12.5)
Diuretics	10 (8.9)
ARBs	9 (8)
Metformin	7 (6.2)
Glimepiride	7 (6.2)
Gliclazide	5 (4.5)
cT	
1	1 (0.9)
2	10 (8.9)
3	99 (88.4)
4	2 (1.8)
cN+	91 (81.2)
Distance to the anal verge (cm)	
0–5	42 (37.5)
6–10	52 (46.4)
11–15	18 (16.1)
Neoadjuvant	
CRT	27 (24.1)
RT	85 (75.9)
Time RT-S > 6 weeks	45 (40.2)
Loop ileostomy	23 (20.5)
ypG	
1	5 (4.5)
2	82 (73.2)
3	8 (7.1)
x	17 (15.2)
ypT	
0	1 (0.9)
2	20 (17.9)
3	91 (81.2)
TRG	
0–1	27 (24.1)
2–3	85 (75.9)
Mucinous component	6 (5.4)
Tumour deposits	29 (25.9)
PLN count	3.59 (3.75)*
NLN count	10.69 (6.16)*
LNY	13.99 (6.79)*
ypN	
1	79 (70.5)
2	33 (29.5)
LNR	0.26 (0.21)*
LODDS	− 1.3 (1.18)*
ENE	14 (12.5)
LVI	10 (8.9)
PNI	13 (11.6)
Distal margin (cm)	2.11 (1.61)*
Adjuvant CT	89 (79.5)
Adjuvant CT > 3 cycles	78 (69.6)

*BMI* body mass index, *CAD* coronary artery disease, *AH* arterial hypertension, *CCI* Charlson comorbidity index, *ACEIs* angiotensin-converting enzyme inhibitors, *ARBs* angiotensin receptor blockers, *CRT* chemoradiotherapy, *RT* radiotherapy, *Time RT-S* time from radiotherapy completion to surgery, *G* histological tumour grade, *TRG* tumour regression grade, *PLN* positive lymph nodes, *NLN* negative lymph nodes, *LNY* lymph node yield, *LNR* lymph node ratio, *LODDS* log odds of positive lymph nodes, *ENE* extranodal extension, *LVI* lymphovascular invasion, *PNI* perineural invasion, *CT* chemotherapy, *SD* standard deviation

^*^Continuous variable

### Procedures

All the patients received neoadjuvant therapy: radiotherapy (RT) at a total dose of 25–42 Gy or chemotherapy (CRT) at a dose of 42–54 Gy combined with one or two cycles of chemotherapy based on 5-fluorouracil. Before surgery, mechanical bowel preparation was performed with the administration of an oral antibiotic and perioperative intravenous antibiotic prophylaxis. AR was performed using laparotomy with total mesorectal excision. End-to-end anastomosis of bowel was performed with a circular stapler. AL, in accordance with the International Study Group of Rectal Cancer, was defined as a deficit at the anastomotic site leading to a communication between the intra- and extraluminal compartments and/or presence of pelvic abscess near the anastomosis [10]. AL was qualified as early if diagnosed within 30 days of surgery, and as late if occurred after that time. Adjuvant treatment was based on 5-fluorouracil. The histopathological examination was based on standard methods of searching for lymph nodes in the surgical specimen. Tumour regression grade (TRG) was based on the assessment of the degree of fibrosis compared to the residual tumor tissue and ranged from 0 to 3, i.e. 0 (complete response), 1 (< 10% residual tumor), 2 (10–50%) and 3 (> 50%).

### Variables

Staging was assessed on the basis of the American Joint Committee on Cancer, TNM Staging System, 8th edition, 2017. LNR was calculated as the PLN to LNY ratio, while LODDS was calculated with the formula ln[(PLN count)/(NLN count)]. In the LODDS and LNR analysis in ypN1c patients, the PLN count was treated as no data and was excluded from this part of the analysis. Additional potential risk factors subject to analysis included: age, sex, body mass index (BMI), presence of comorbidities, CCI, medications used, type of neoadjuvant therapy (RT vs. CRT), time from RT completion to surgery, clinical staging of the disease before treatment, tumour distance from the anal verge, presence of loop ileostomy, occurrence of postoperative complications, TRG, perineural invasion (PNI), lymphovascular invasion (LVI), extranodal extension (ENE), width of distal margin, adjuvant chemotherapy.

### Statistical methods

The effect of potential factors on survival was assessed with the use of Kaplan–Meier curves together with a log-rank test and Cox proportional hazards model. The estimation of cut-off points for the parameters related to nodal staging was based on the analysis of Kaplan–Meier curve difference significance for iteratively increased cut-off thresholds. All calculations were made using the statistical package R version 3.5.3.

## Results

In the study group, 3- and 5-year disease-free survival (DFS) was 71.9% and 59.7%, and disease-specific survival (DSS) was 85.5% and 74.4%, respectively. The mean follow-up period in the study group was 57 months. Loop ileostomy during the primary procedure was created in 23/112 (20.5%) of patients. Postoperative complications were observed in 39/112 (34.8%) of patients. AL was observed in 23/112 (20.5%) of patients, including 16/23 (69.6%) early and 7/23 (30.4%) late ALs. In 15/23 (65.2%) cases of AL, anastomosis was separated by performing the Hartmann’s procedure. Aside to the above, abnormal wound healing was observed in 6 patients, and there were 3 cases of urinary tract infection, 3 cases of pneumonia, 3 cases of bleeding and 1 case of mechanical obstruction. Postoperative complications are presented in the Additional file 1. 19 (17%) patients used angiotensin-converting enzyme inhibitors (ACEIs) at the time of treatment initiation, with 5 patients using ramipril, 5 perindopril, 3 enalapril, 2 lisinopril, 2 trandolapril, 1 cilazapril and 1 imidapril. 9 (8%) patients used angiotensin receptor blockers (ARBs).

### Analysis of nodal staging parameters

For the LNY variable, no cut-off point for which Kaplan–Meier curves would significantly differ was found. Similarly, no differences in survival were found while comparing nodal staging (ypN1 vs. ypN2) according to the TNM classification. For the NLN count, significant differences in survival were achieved for the cut-off point 5 (≤ 5 vs. > 5) for DSS (*p* = 0.0045) and the cut-off point 7 (≤ 7 vs. > 7) for DFS (*p* = 0.029). Results of the analysis of nodal staging parameters are presented in the Additional file 2.

### Analysis of survival

Based on Kaplan–Meier curves and a log-rank test, we did not reveal an effect of comorbidities on survival, both with regard to separate analysis, and that based on CCI.

**Fig. 2:**
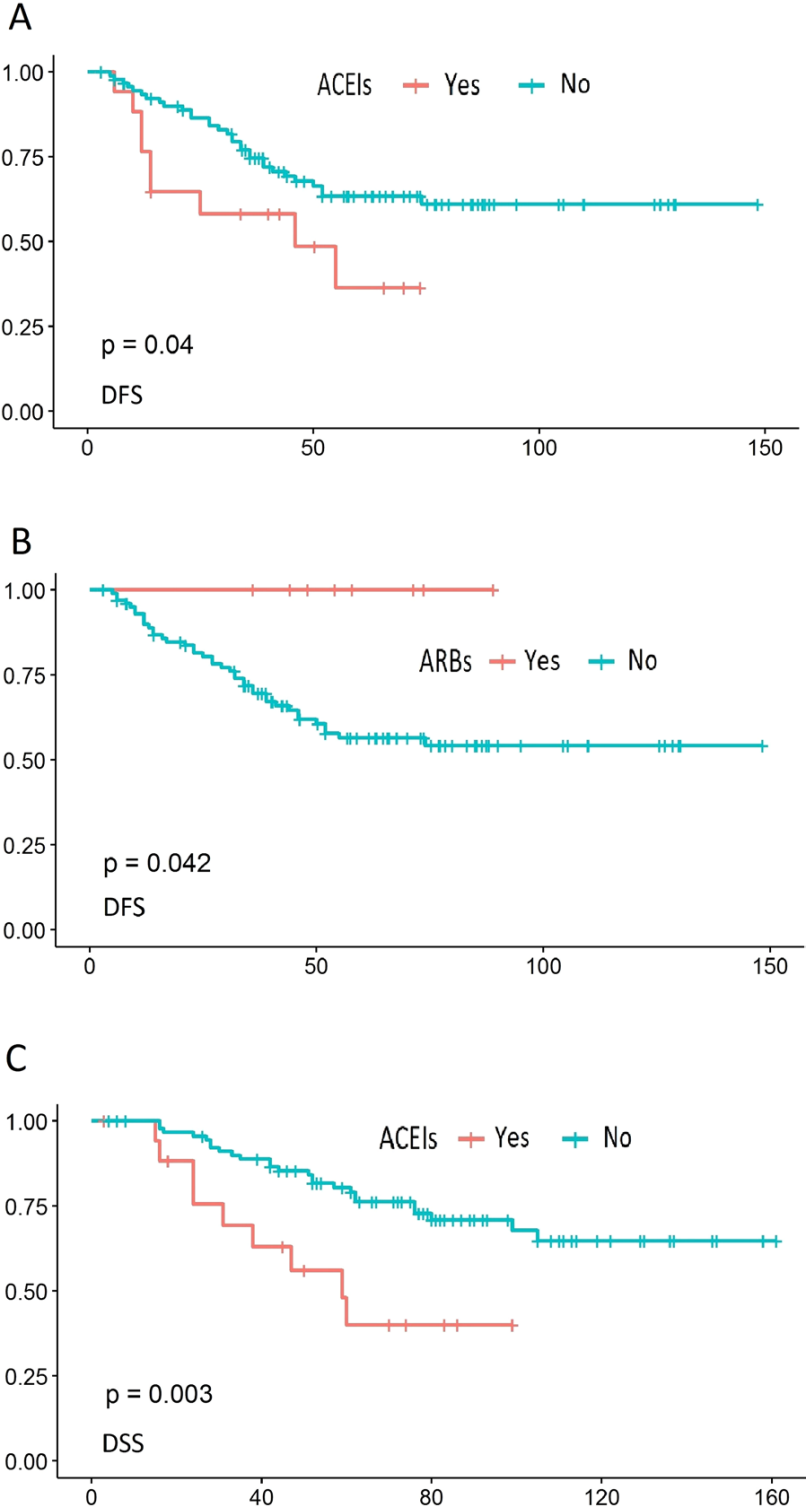
Survival analysis (DFS) of patients depending on angiotensin-converting enzyme inhibitors (**A**), DFS depending on angiotensin receptor blockers (**B**) and DSS depending on angiotensin-converting enzyme inhibitors (**C**)

A negative effect of the use of ACEIs (*p* = 0.04) (Fig. 2a) and metformin (*p* = 0.048), and a positive effect of the use of ARBs (*p* = 0.042) (Fig. 2b) on DFS was shown. In addition, a negative effect of the occurrence of complications, regardless of the degree in the Clavien–Dindo classification (*p* = 0.012), occurrence of AL (*p* = 0.024), and after dividing AL into early and late, of early AL (*p* = 0.0095) on DFS was shown. Histological grade G3 (*p* = 0.02) and the presence of PNI (*p* = 0.00015) had a negative effect on DFS, as well.

The DSS analysis showed an adverse effect of the use of ACEIs (*p* = 0.003) (Fig. 2c), metformin (*p* = 0.016), occurrence of complications, regardless of the degree in the Clavien–Dindo classification (*p* = 0.025), occurrence of AL (*p* = 0.0049), and after dividing AL into early and late, of early AL (*p* = 0.00027), and histological grade G3 (*p* = 0.018). No effect of other analysed factors on survival was revealed, including the effect of neoadjuvant treatment regimen (see Additional file 3).

### Cox proportional hazards model

Results of Cox analysis are shown in Table 2. For DFS in a multiple factor analysis, significant adverse factors were: the use of ACEIs (HR: 3.11, 95% CI 1.01–9.56, *p* = 0.047), presence of PNI (HR: 7.27, 95% CI 2.74–19.3, *p* < 0.001), and the occurrence of postoperative complications (HR: 6.79, 95% CI 2.09–22.11, *p* = 0.001). And a positive factor was the NLN count > 7 (HR: 0.33, 95% CI 0.12–0.88, *p* = 0.026). In the DSS analysis, an adverse factor was the use of ACEIs (HR: 4.275, 95% CI 1.44–12.694, *p* = 0.009), while a positive effect was caused by NLN > 5 (HR: 0.22, 95% CI 0.082–0.586, *p* = 0.002). The other analysed factors were not significant in the multiple factor analysis. The above multivariate Cox analysis models were then further analyzed using the likelihood ratio test. The course and the results of this analysis are given in Additional file 4.

**Table 2:** Cox proportional hazards model

	Single factor analysis			Multiple factor analysis		
	HR	95% CI	*p*	HR	95% CI	*p*
*Disease-specific survival (DSS)*						
Sex: male	0.98	0.49–1.96	0.958			
Age > 65 years	1.41	0.72–2.77	0.321			
BMI > 30	1.32	0.60–2.93	0.495			
AH	1.63	0.83–3.21	0.154			
Diabetes mellitus	1.25	0.52–3.01	0.625			
CCI > 2	0.86	0.37–1.97	0.716			
Metformin	3.41	1.18–9.83	0.023	1.42	0.32–6.22	0.644
Alpha blockers	0.99	0.14–7.25	0.992			
Beta blockers	1.54	0.74–3.23	0.250			
ACEIs	3.05	1.41–6.60	0.005	4.28	1.44–12.69	0.009
CCBs	2.14	0.88–5.17	0.093			
Diuretics	0.95	0.29–3.11	0.930			
Noeadjuvant CRT	Ref					
Neoadjuvant RT	1.16	0.50–2.68	0.731			
RT-S ≥ 6 weeks	1.02	0.50–2.06	0.963			
Distance to the anal verge 1–5 cm	Ref					
6–10 cm	1.67	0.78–3.57	0.186			
11–15 cm	1.17	0.37–3.75	0.787			
Loop ileostomy	1.30	0.59–2.87	0.518			
Complications	2.12	1.08–4.16	0.029	2.18	0.47–10.07	0.317
ypT0-2	Ref					
ypT3	1.16	0.48–2.80	0.745			
LNY ≥ 12	0.68	0.35–1.34	0.266			
ypG3	3.01	1.14–7.94	0.026	2.82	0.69–11.52	0.149
ENE	0.98	0.35–3.75	0.968			
LVI	1.19	0.36–3.89	0.777			
PNI	1.79	0.69–4.63	0.230			
TRG 0–1	Ref					
TRG 2–3	1.34	0.58–3.08	0.496			
CT > 3cycles	0.58	0.28–1.20	0.140			
NLN > 5	0.36	0.17–0.75	0.006	0.22	0.08–0.59	0.002
PLN > 11	3.47	1.05–11.46	0.041	2.65	0.45–15.44	0.280
LNR > 0.15	2.18	1.01–4.73	0.048	1.15	0.13–10.19	0.899
*Disease-free survival (DFS)*						
Sex:male	1.09	0.58–2.07	0.788			
Age > 65 years	1.01	0.53–1.89	0.993			
BMI > 30	1.13	0.54–2.37	0.752			
AH	1.35	0.72–2.51	0.349			
Diabetes mellitus	1.21	0.54–2.75	0.642			
CCI > 2	0.72	0.32–1.63	0.429			
Metformin	2.76	0.97–7.83	0.006	0.85	0.22–3.26	0.808
Alpha blockers	1.13	0.16–8.24	0.904			
Beta blockers	1.59	0.81–3.12	0.181			
ACEIs	2.15	1.02–4.55	0.044	3.11	1.01–9.56	0.047
CCBs	1.98	0.87–4.48	0.103			
Diuretics	1.16	0.41–3.27	0.776			
Neoadjuvant RT	1.09	0.52–2.29	0.824			
RT-S ≥ 6 weeks	0.79	0.41–1.49	0.463			
Distance to the anal verge 1–5 cm	Ref					
6–10 cm	1.35	0.68–2.69	0.390			
11–15 cm	0.95	0.34–2.66	0.916			
Loop ileostomy	1.21	0.58–2.54	0.614			
Complications	2.18	1.17–4.05	0.014	6.79	2.09–22.11	0.001
ypT0-2	Ref					
ypT3	1.48	0.62–3.54	0.374			
LNY ≥ 12	0.92	0.49–1.73	0.796			
ypG3	2.69	1.12–6.48	0.028	0.75	0.13–4.21	0.748
ypN1	Ref					
ypN2	1.02	0.52–2.00	0.961			
ECI	1.25	0.53–2.98	0.613			
LVI	1.79	0.70–4.57	0.225			
PNI	4.09	1.86–8.97	0.001	7.27	2.74–19.30	< 0.001
TRG 0–1	Ref					
TRG 2–3	1.27	0.59–2.76	0.545			
CT > 3cycles	0.82	0.41–1.64	0.569			
NLN > 7	0.50	0.26–0.94	0.032	0.33	0.12–0.88	0.026
PLN > 10	2.72	1.06–6.99	0.038	0.76	0.20–2.95	0.692
LNR > 0.3	1.89	1.00–3.60	0.052	1.20	0.25–5.84	0.821
LODDS > − 1	1.89	1.00–3.58	0.051	1.25	0.29–5.51	0.766

*HR* hazard ratio, *CI* confidence interval, *DFS* disease-free survival, *DSS* disease-specific survival, AH-arterial hypertension, *CCI* Charlson comorbidity index, *ACEIs* angiotensin-converting enzyme inhibitors, *CCBs* calcium channel blockers, *RT-S* time from completion of radiotherapy to surgery, *PLN* positive lymph nodes, *NLN* negative lymph nodes, *LNR* lymph node ratio, *LODDS* log odds of positive lymph nodes, *LNY* lymph node yield, *G* histological tumour grade, *ENE* extranodal extension, *LVI* lymphovascular invasion, *PNI* perineural invasion, *TRG* tumour regression grade, *CT* adjuvant chemotherapy

## Discussion

The study showed a negative effect of the use of ACEIs and low NLN count on both DSS, and DFS. Additionally, DFS was negatively influenced by the presence of PNI and the occurrence of postoperative complications.

We showed a negative effect of ACEIs on survival independently of comorbidities, analysed both separately and based on CCI. The renin-angiotensin system (RAS) is one of the phylogenetically oldest endocrine systems, whose main task is to regulate water and sodium metabolism. The main component of this system is angiotensin II (AngII), which is developed from angiotensin I (AngI) with the participation of angiotensin converting enzyme (ACE) [11]. Aside to RAS in the circulatory system, a presence of all components of this system was shown in tissues (tissue RAS–tRAS), including in the neoplastic cells and tumour microenvironment cells, such as tumour associated macrophages, regulatory T cells and fibroblasts [12, 13]. This system acts in two major mechanisms opposite to each other through the AngII type 1 receptor (AT1R) and AngII type 2 receptor (AT2R). Activation of the ACE/AngII/AT1R pathway leads to vasoconstriction with consequential acidosis and hypoxia, which triggers expression of proinflammatory cytokines. Additionally, angiogenesis is intensified by an increased expression of the vascular endothelial growth factor, increase in tumour progression and its metastatic potential. And the activation of the AT2R/ACE2/Ang1-7/MasReceptor pathway triggers an antiinflammatory, antiproliferative, vasodilating and antiangiogenic effect [14]. It is estimated that about 40% AngII is formed in ACE-independent pathways [15]. Chymase, which is a serine protease, shows ACE-like activity and the ability to convert AngI to AngII. Under physiological conditions, it occurs in the form of inactive pro-chymase, and its activation is increased in the setting of local inflammation and oxidative stress [16]. Immunohistochemistry tests of tumour specimens collected from patients with colorectal cancer showed expression of chymase in the mast cells in the tumour microenvironment. It was also found that such expression was significantly higher in the group of subjects with distant metastases versus the group free of metastases [17]. Studies of HT-29 cell lines of colorectal cancer showed that these cells did not show ACE expression while showing AT1R and chymase expression. Moreover, it was shown that ACEIs did not affect AngII production in HT-29 cells [18]. Therefore, it seems that tRAS activation by AT1R in colorectal cancer may occur independently of ACE. In the light of the above data it was shown that currently there is no theoretical basis to claim that the use of ACEIs may have a significant inhibitory effect on the tRAS cascade in the colorectal tumour.

Close interactions were shown between RAS and kallikrein-kinin system (KKS) at several levels [19]. Bioactive kinins, as well as bradykinin (BK) and kallidin are formed from kininogens with the participation of plasma and tissue kallikreins. On the other hand, BK, triggered by kinases, including ACE, is converted to active des-Arg9-BK and inactive peptides. KKS affects cells with the use of two types of kinin receptors, type 1 (B1R), whose ligand is des-Arg9-BK, and type 2 (B2R), whose ligand is BK. B2R shows high expression in various tissues, while B1R is almost undetectable under physiological conditions, and its expression increases under the influence of proinflammatory cytokines, in the setting of inflammation and tissue damage. The exception are fibroblasts, which show constitutive expression of B1R [20]. There is evidence that kinins play an important role in the recruitment of proinflammatory cells in the tumour microenvironment, and stimulate neoplastic cell migration and invasion, thus increasing their metastatic potential [21, 22]. Studies of human and murine cell lines of colorectal cancer, including HT-29, revealed high expression of both B1R and B2R [23, 24]. Wang et al. showed that blocking of B2R weakens the process of invasion and migration of colorectal cancer cells [23]. Also da Costa et al. showed expression of both B1R and B2R in murine (MoCR) and human (SW-480) cell lines of colorectal cancer. In addition, in vivo and in vitro models revealed that blocking of kinin receptors inhibits tumour growth and reduces its metastatic potential [25]. It was also shown that kinins stimulate the process of angiogenesis, both in normal and cancerous cells [26]. ACE, aside to converting AngI to AngII, degrades BK to inactive peptides by removing two C-terminal amino acids. The use of ACEIs was shown to reduce plasma AngII and at the same time to increase the level of B2R agonists [27]. This also leads to a situation of increased level of their des-Arg metabolites, which are B1R agonists. Thus, the use of ACEIs may indirectly lead to an increased activation of KKS, both via B2R and B1R [28]. ACEIs were also shown to be allosteric enhancers of kinin receptors and to have a direct effect on the enhancement of the signal transmitted by their pathway [28]. The issue of possible AT1R/B2R heterodimer formation remains unclear, since reports on this matter are contradictory [29, 30].

The effect of using ACEIS on the interactions between tRAS and KKS in the aspect of their influence on colorectal cancer progression is poorly known. However, summing up, it may be concluded that despite the use of ACEIs, it is possible to activate the procancerous tRAS pathway via AT1R in the neoplastic tumour with the use of chymase. Additionally, local inflammatory process in the tumour microenvironment leads to an increased B1R expression, and the use of ACEIs may lead to increased KKS activation, both via B1R and B2R. This takes place in two mechanisms: inhibiting the kinin breakdown with consequential increase in the level of both receptor agonists, and allosteric enhancement of the transmitted signal. These interactions may lead to a negative effect of ACEIs on the course of the neoplastic disease, and explain our results.

There are few reports on the effect of RASIs on treatment outcomes in patients with rectal cancer. Typically, publications discuss subjects with colorectal cancer. Additionally, the effect of both groups of RASIs is assessed jointly (ACEIs/ARBs), which may significantly affect the results and cause conflicting conclusions. The effect of these drugs on the rectal cancer response to neoadjuvant treatment is unclear. Morris et al. showed that the use of ACEIs/ARBs significantly increases the frequency of complete responses after preoperative radiotherapy in a multiple factor analysis [3], while Rombouts et al. did not confirm in their study a positive effect of these drugs [31]. Similar discrepancies exist regarding the assessment of the ACEIs/ARBs effect on long-term survival. In a retrospective analysis of nearly 14,000 patients performed on the basis of SEER database, Balkrishnan et al. showed that better indices of cancer-specific mortality were shown by patients with colorectal cancer stage I–III, who received antihypertensive drugs, as compared to patients who did not receive such drugs. A detailed analysis showed a positive effect on survival of all study drug groups (ACEIs, beta blockers, diuretics), except for ARBs. Due to data availability, the analysis excluded, however, patients below 65 years old, and those using antihypertensive drugs before the diagnosis of colorectal cancer [32]. On the other hand, Holmes et al. and Cardwell et al. in two population studies, showed no such correlation in other groups of patients with colorectal cancer (n = 3967 and n = 4762, respectively) [33, 34]. At the same time it was shown that the use of ACEIs/ARBs may increase the risk of death in the course of breast cancer and lung cancer [33]. Ozawa et al. showed a positive effect of ACEIs/ARBs on DFS in the course of left-sided colorectal cancer and stage I cancer. Both groups of drugs were analysed jointly, and the authors did not specify how many patients used ARBs, and how many used ACEIs [35]. In a retrospective analysis of 262 patients with colorectal cancer, Engineer et al. showed a positive effect of the use of RASIs + beta blockers. However, this publication does not show the ACEIs to ARBs ratio in the study group, either [36]. In an earlier study performed at the same institution, Heinzerling et al. showed a positive effect of the use of ACEIs on the occurrence of distant metastases in subjects with stage II colorectal cancer in a retrospective analysis of 55 patients. This study, however, has significant limitations. ACEIs are not directly included in the statistical analysis. Conclusions on their effect are made indirectly based on the frequency of their use by patients with arterial hypertension, which was a significant factor in the multiple factor analysis [37]. Dai et al. showed in their meta-analysis, that although the ACEIs/ARBs therapy may be related to a reduced incidence of colorectal cancer, there is no evidence that it may also affect treatment outcomes of patients with colorectal cancer [38].

To the best of our knowledge, the effect of ACEIs has not been assessed so far exclusively in the group of subjects with ypN+ rectal cancer. The analysis results point to a need of further studies in this group of patients. If our results are confirmed with larger, independent groups of patients, the exclusion of ACEIs from the therapy of comorbidities could be a simple method of improving long-term oncological outcomes in patients with rectal cancer.

We have shown a negative effect of postoperative complications on DFS, regardless of the degree in the Clavien–Dindo classification. Similar conclusions were drawn by Sprenger et al. who showed that the occurrence of any surgical complications (anastomotic leakage and/or abnormal wound healing) had a significant negative effect on OS and local recurrence free survival among the patients of the German Rectal Cancer Trial, as shown by a multiple factor analysis [39]. Possible mechanisms underlying the effect of complications on long-term oncological outcomes include no or delayed adjuvant therapy [40]. In a post hoc analysis we showed that the patients with complications significantly more often failed to receive > 3 cycles of adjuvant chemotherapy, which could suggest that such a claim is correct. In a multiple factor analysis, however, we confirmed the reports of no positive effect on DFS and DSS of adjuvant fluoropyrimidine-based chemotherapy used in the analysed period [41, 42].

The only parameter related to lymph nodes which in our analysis had a significant effect on survival was the NLN count in the surgical specimen, which is confirmed by observations of several authors [7, 43]. There have been promising attempts, yet requiring validation, to modify the current AJCC classification by adding the NLN count parameter [44]. This effect is explained by some authors by the presence of small (up to 2 mm) lymph nodes containing micrometastases which are not detected in standard HE staining and for that reason are assessed as NLN by pathologists. It is believed that an increased NLN count may reduce the risk of their non-removal, and, as a result, disease relapse [45]. Another possible explanation is an increase in the NLN count resulting from a stronger immune response to the tumour, with accompanying reactive lymph node enlargement. This phenomenon has a positive prognostic value and facilitates finding a higher number of lymph nodes in the specimen [46, 47].

The analysis has typical limitations of retrospective and single-centre analyses. The neoadjuvant treatment was not performed with the use of a uniform schedule. However, we have shown no effect of this factor on the treatment outcomes. Data on comorbidities and medications taken were obtained from internal and anaesthesiological consultation records prior to surgery. It was not possible to assess the duration of using the medications.

## Conclusions

The use of ACEIs may have a negative effect on long-term treatment outcomes in patients with ypN + M0 rectal cancer. In this group of patients, the NLN count seems to be an important prognostic factor, as well.

## Supplementary Information

**Additional file 1**. Postoperative complications.

**Additional file 2**. Results of the analysis of nodal staging parameters.

**Additional file 3**. Survival analysis depending on neoadjuvant treatment regimen.

**Additional file 4**. Likelihood ratio test.

**Additional file 5**. Dataset.

## Data Availability

The dataset supporting the conclusions of this article is included within the article (Additional file 5).

## Abbreviations

ACE: Angiotensin-converting enzyme
ACEIs: Angiotensin-converting enzyme inhibitors
AL: Anastomotic leakage
AngI: Angiotensin I
AngII: Angiotensin II
AR: Anterior resection
ARBs: Angiotensin receptor blockers
AT1R: Angiotensin II type 1 receptor
AT2R: Angiotensin II type 2 receptor
BK: Bradykinin
B1R: Kinin B1 receptor
B2R: Kinin B2 receptor
BMI: Body mass index
CAD: Coronary artery disease
CCI: Charlson comorbidity index
CRT: Chemoradiotherapy
CI: Confidence interval
DFS: Disease-free survival
DSS: Disease-specific survival
ENE: Extranodal extension
G: Histological tumour grade
HR: Hazard ratio
KKS: Kallikrein-kinin system
LNR: Lymph node ratio
LNY: Lymph node yield
LODDS: Log odds of positive lymph nodes
LVI: Lymphovascular invasion
NLN: Negative lymph nodePLN- positive lymph node
PNI: Perineural invasion
RAS: Renin-angiotensin system
RASIs: Renin-angiotensin system inhibitors
RT: Radiotherapy
SD: Standard deviation
t-RAS: Tissue renin-angiotensin system
TRG: Tumour regression grade
