# Supplementary material for: Risk factors for long-term survival in patients with ypN+ M0 rectal cancer after radical anterior resection
Source: BMC Gastroenterol. 2022 Mar 26;22:141. doi: 10.1186/s12876-022-02226-9 (PMC8961971; doi:10.1186/s12876-022-02226-9)
Supplement: Supplementary file 1 — Additional file 1. Postoperative complications. [file 12876_2022_2226_MOESM1_ESM.docx]

|  |  | n (%) |
| --- | --- | --- |
| Complications (Clavien-Dindo) | 0 | 73 (65.2) |
|  | 1-2 | 14 (12.5) |
|  | >2 | 25 (22.3) |
| AL | Early AL | 16 (14.3) |
|  | Late AL | 7 (6.2) |
| Abnormal wound healing |  | 6 (5.4) |
| Urinary tract infection |  | 3 (2.7) |
| Pneumonia |  | 3 (2.7) |
| Postoperative bleeding |  | 3 (2.7) |
| Ileus |  | 1 (0.9) |

Additional file 1

Title: Postoperative complications

AL- anastomotic leakage
