# Supplementary material for: Risk factors for long-term survival in patients with ypN+ M0 rectal cancer after radical anterior resection
Source: BMC Gastroenterol. 2022 Mar 26;22:141. doi: 10.1186/s12876-022-02226-9 (PMC8961971; doi:10.1186/s12876-022-02226-9)
Supplement: Supplementary file 2 — Additional file 2. Results of the analysis of nodal staging parameters. [file 12876_2022_2226_MOESM2_ESM.docx]

Additional file 2

Title: Survival analysis depending on lymph node parameters


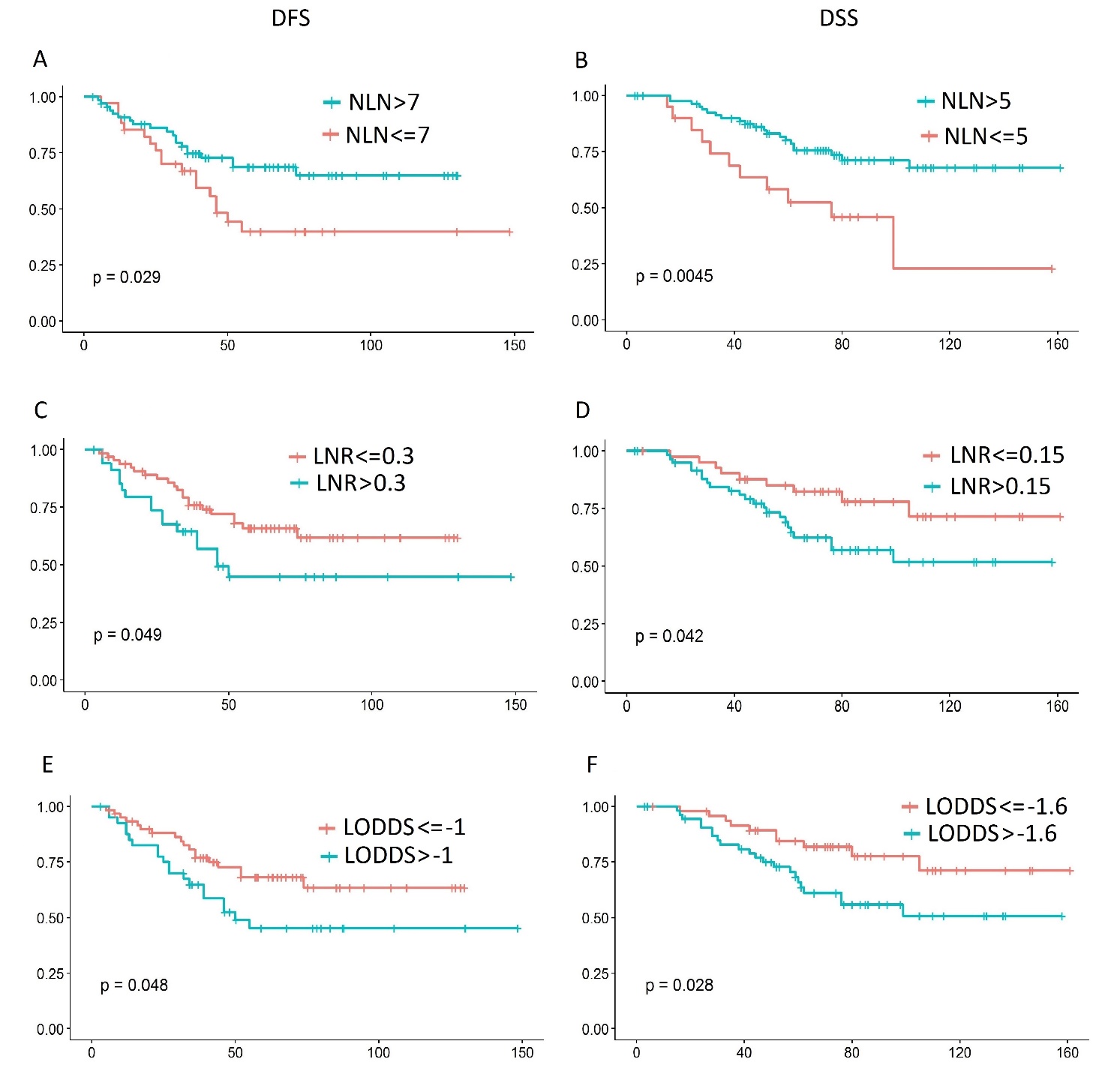


Survival analysis (DFS and DSS) depending on negative lymph node count (NLN), lymph node ratio (LNR) and log odds of positive lymph nodes (LODDS). For the PLN count, significant differences in survival were found only at the cut-off point 11 (<= 11 vs. > 11) for DSS (p= 0.03), and at the cut-off point for 10 (<= 10 vs. > 10) for DFS (p= 0.033). For the NLN count, significant differences in survival were achieved for the cut-off point 5 (<= 5 vs. > 5) for DSS (p= 0.0045) and the cut-off point 7 (<= 7 vs. > 7) for DFS (p= 0.029) (A, B). For LNR, significant differences in survival were shown for the cut-off point 0.3 (<=0.3 vs. >0.3) for DFS (p=0.049), and for the cut-off point 0.15 (<= 0.15 vs. > 0.15) for DSS (p= 0.042) (C, D). For LODDS, significant differences in survival were achieved for the cut-off point -1 (<= -1 vs > -1) for DFS (p= 0.048), and for the cut-off point -1.6 (<= -1.6 vs. > -1.6) for DSS (p= 0.028) (E, F).
