# Supplementary material for: Risk factors for long-term survival in patients with ypN+ M0 rectal cancer after radical anterior resection
Source: BMC Gastroenterol. 2022 Mar 26;22:141. doi: 10.1186/s12876-022-02226-9 (PMC8961971; doi:10.1186/s12876-022-02226-9)
Supplement: Supplementary file 3 — Additional file 3. Survival analysis depending on neoadjuvant treatment regimen. [file 12876_2022_2226_MOESM3_ESM.docx]

Additional file 3

Title: The effect of neoadjuvant treatment on survival


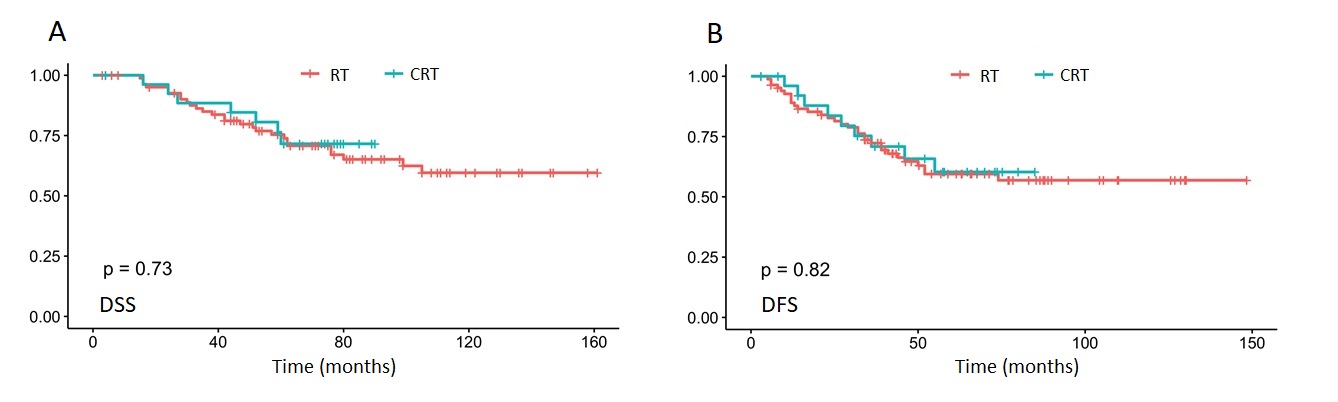


The effect of neoadjuvant radiotherapy (RT) vs. chemoradiotherapy (CRT) on disease-specific survival (A) and disease-free survival (B).
