# Supplementary material for: Risk factors for long-term survival in patients with ypN+ M0 rectal cancer after radical anterior resection
Source: BMC Gastroenterol. 2022 Mar 26;22:141. doi: 10.1186/s12876-022-02226-9 (PMC8961971; doi:10.1186/s12876-022-02226-9)
Supplement: Supplementary file 4 — Additional file 4. Likelihood ratio test. [file 12876_2022_2226_MOESM4_ESM.docx]

**Likelihood ratio (LR) test**

For the previously selected multivariate models, we analyzed how the LR test result would change when removing the next statistically insignificant variables from the model. The variables were removed starting from those for which the highest value of the LR test was obtained. The following hypotheses were tested each time for the model with the removal of selected variables:

*H_0_*: the model with the variable removed fits the data equally well as the full model. Therefore, we should use the model with the variable removed;

*H_1_*: the full model is a better fit to the data compared to the model with the variable removed. Therefore, we should use the full model.

**a/Disease-specific survival (DSS)**

Full multivariate Cox regression model for DSS

|  | HR | 2.5 % | 97.5 % | P |
| --- | --- | --- | --- | --- |
| NLN > 5 | 0.220 | 0.082 | 0.586 | 0.002 |
| ypG3 | 2.819 | 0.690 | 11.518 | 0.149 |
| PLN > 11 | 2.646 | 0.453 | 15.443 | 0.280 |
| Metformin | 1.418 | 0.323 | 6.217 | 0.644 |
| LNR > 0.15 | 1.151 | 0.130 | 10.189 | 0.899 |
| ACEIs | 4.275 | 1.440 | 12.694 | 0.009 |
| Complications | 2.182 | 0.473 | 10.068 | 0.317 |

The analysis started by removing the ypG variable for which the value of the LR test was the highest. The model without this variable was estimated, and then it was compared whether the model with the variable removed was as well fitted to the data as the full model. The obtained value of the LR test was p=0.003 < 0.05. Therefore, it is necessary to reject the null hypothesis and conclude that the full model is a better fit to the data than the model with the removed ypG variable.

Then the LNR>0.15 variable was removed from the model - the value of the LR test reached p=0.799>0.05. The variable was removed from further analysis.

Next, the Metformin variable was removed from the model and the models were compared one more time - the value of the LR test reached p=0.631>0.05. The variable was removed from further analysis.

Next, the PLN>11 variable was removed from the model - the value of the LR test reached p=0.286>0.05. The variable was removed from further analysis.

Multivariate Cox regression model for DSS obtained after conducting LR tests

|  | HR | 2.5 % | 97.5 % | P |
| --- | --- | --- | --- | --- |
| NLN > 5 | 0.187 | 0.082 | 0.428 | <0.001 |
| ypG3 | 3.190 | 0.875 | 11.622 | 0.079 |
| Complications | 4.876 | 2.175 | 10.929 | <0.001 |
| ACEIs | 4.669 | 1.695 | 13.024 | 0.003 |

**b/ Disease-free survival (DFS)**

Full multivariate Cox regression model for DFS

|  | HR | 2.5 % | 97.5 % | P |
| --- | --- | --- | --- | --- |
| NLN > 7 | 0.329 | 0.124 | 0.876 | 0.026 |
| ypG3 | 0.754 | 0.135 | 4.208 | 0.748 |
| PLN > 10 | 0.760 | 0.196 | 2.947 | 0.692 |
| Metformin | 0.846 | 0.219 | 3.261 | 0.808 |
| LNR > 0.3 | 1.201 | 0.247 | 5.836 | 0.821 |
| LODDS > -1 | 1.252 | 0.285 | 5.507 | 0.766 |
| ACEIs | 3.112 | 1.013 | 9.556 | 0.047 |
| Complications | 6.790 | 2.085 | 22.113 | 0.001 |
| PNI | 7.266 | 2.736 | 19.297 | <0.001 |

The analysis started by removing the ypG variable. The obtained value of the LR test was p=0.003<0.05. Therefore, the null hypothesis should be rejected and it should be concluded that the full model is a better fit to the data than the model with the removed ypG variable.

The LNR variable was then removed from the model - the value of the LR test reached p=0.819>0.05. This variable was removed from further analysis.

The Metformin variable was then removed from the model and compared with the existing model. The value of the LR test reached p=0.802>0.05. This variable was removed from further analysis.

Next, the PLN>10 variable was removed from the model. The value of the LR test reached p=0.757>0.05. This variable was removed from further analysis.

Next, the LODDS > -1 variable was removed from the model. The value of the LR test reached p=0.426>0.05. This variable was removed from further analysis.

Another variable, ACEIs, was then removed from the model and compared with the existing model. The value of the LR test reached p=0.069>0.05. This variable was removed from further analysis.

Multivariate Cox regression model for DFS obtained after conducting LR tests.

|  | HR | 2.5 % | 97.5 % | p |
| --- | --- | --- | --- | --- |
| ypG3 | 1.512 | 0.516 | 4.428 | 0.450 |
| Complications | 4.061 | 2.002 | 8.239 | <0.001 |
| PNI | 6.916 | 2.837 | 16.860 | <0.001 |
| NLN > 7 | 0.293 | 0.140 | 0.611 | 0.001 |

HR- hazard ratio, DFS- disease-free survival, DSS- disease-specific survival, ACEIs- angiotensin-converting enzyme inhibitors, PLN- positive lymph nodes, NLN- negative lymph nodes, LNR- lymph node ratio, LODDS- log odds of positive lymph nodes, ypG- histological tumour grade, PNI- perineural invasion
